# The adaptor protein PID1 regulates receptor-dependent endocytosis of postprandial triglyceride-rich lipoproteins

**DOI:** 10.1016/j.molmet.2018.07.010

**Published:** 2018-07-30

**Authors:** Alexander W. Fischer, Kirstin Albers, Lucia M. Krott, Britta Hoffzimmer, Markus Heine, Hartwig Schmale, Ludger Scheja, Philip L.S.M. Gordts, Joerg Heeren

**Affiliations:** 1 Department of Biochemistry and Molecular Cell Biology, University Medical Center Hamburg-Eppendorf, 20246, Hamburg, Germany; 2 Department of Medicine, University of California, La Jolla, San Diego, CA, 92093, USA

**Keywords:** Lipid metabolism, Insulin, Adaptor proteins, Lipoprotein receptors, Endocytosis, Atherosclerosis

## Abstract

**Objective:**

Insulin resistance is associated with impaired receptor dependent hepatic uptake of triglyceride-rich lipoproteins (TRL), promoting hypertriglyceridemia and atherosclerosis. Next to low-density lipoprotein (LDL) receptor (LDLR) and syndecan-1, the LDLR-related protein 1 (LRP1) stimulated by insulin action contributes to the rapid clearance of TRL in the postprandial state. Here, we investigated the hypothesis that the adaptor protein phosphotyrosine interacting domain-containing protein 1 (PID1) regulates LRP1 function, thereby controlling hepatic endocytosis of postprandial lipoproteins.

**Methods:**

Localization and interaction of PID1 and LRP1 in cultured hepatocytes was studied by confocal microscopy of fluorescent tagged proteins, by indirect immunohistochemistry of endogenous proteins, by GST-based pull down and by immunoprecipitation experiments. The *in vivo* relevance of PID1 was assessed using whole body as well as liver-specific *Pid1*-deficient mice on a wild type or *Ldlr*-deficient (*Ldlr*^*−/−*^) background. Intravital microscopy was used to study LRP1 translocation in the liver. Lipoprotein metabolism was investigated by lipoprotein profiling, gene and protein expression as well as organ-specific uptake of radiolabelled TRL.

**Results:**

PID1 co-localized in perinuclear endosomes and was found associated with LRP1 under fasting conditions. We identified the distal NPxY motif of the intracellular C-terminal domain (ICD) of LRP1 as the site critical for the interaction with PID1. Insulin-mediated NPxY-phosphorylation caused the dissociation of PID1 from the ICD, causing LRP1 translocation to the plasma membrane. PID1 deletion resulted in higher LRP1 abundance at the cell surface, higher LDLR protein levels and, paradoxically, reduced total LRP1. The latter can be explained by higher receptor shedding, which we observed in cultured *Pid1*-deficient hepatocytes. Consistently, PID1 deficiency alone led to increased LDLR-dependent endocytosis of postprandial lipoproteins and lower plasma triglycerides. In contrast, hepatic PID1 deletion on an *Ldlr*^*−/−*^ background reduced lipoprotein uptake into liver and caused plasma TRL accumulation.

**Conclusions:**

By acting as an insulin-dependent retention adaptor, PID1 serves as a regulator of LRP1 function controlling the disposal of postprandial lipoproteins. PID1 inhibition provides a novel approach to lower plasma levels of pro-atherogenic TRL remnants by stimulating endocytic function of both LRP1 and LDLR in the liver.

## Highlights

• PID1 is a retention adaptor protein that regulates activity of the endocytic receptor LDL receptor-related protein 1 (LRP1).
• PID1 regulates the insulin-dependent LRP1-mediated endocytosis of lipoproteins *in vivo*.
• PID1 deficiency in liver reduces LRP1 levels via cell surface shedding, and paradoxically increases LDL receptor activity.
• PID1 antagonism has therapeutic potential to reduce pro-atherogenic lipoproteins in hyperlipidemic and diabetic patients.

## 1 Introduction

Disturbances in lipid and lipoprotein metabolism promote the development and progression of atherosclerosis [1], [2]. Next to elevated low-density lipoproteins (LDL), the accumulation of plasma triglyceride-rich lipoproteins (TRL) and their remnants is an important risk factor for cardiovascular disease [3]. TRL are secreted from the liver as very low-density lipoproteins (VLDL) and from the intestine as chylomicrons. Both are processed by lipoprotein lipase (LPL) at the vascular endothelium, mediating the release of free fatty acids that are taken up by adipose tissues and muscles. LPL remains associated after lipolysis and the resulting TRL remnants additionally acquire apolipoprotein E. Both proteins interact with lipoprotein receptors that promote the efficient endocytosis of TRL remnants into hepatocytes [4], [5], [6], a process determining the plasma levels of these pro-atherogenic lipoproteins [7]. In addition to syndecan-1 and the LDLR, the LDLR-related protein 1 (LRP1) is an important endocytic receptor that is especially relevant for the uptake of postprandial chylomicron remnants [5], [8], [9].

One main characteristic of obesity and the Metabolic Syndrome is the accumulation of TRL and their remnants in the circulation [10]. This is explained in part by hepatic and intestinal insulin resistance, which is associated with increased secretion of both VLDL and chylomicrons [10], [11], [12]. In addition, impaired insulin-dependent TRL processing by adipose tissue LPL contributes to postprandial hyperlipidemia [13]. Previously, we demonstrated the relevance of compromised hepatic TRL remnant uptake in insulin resistant mice [14]. Mechanistically, Descamps et al. showed that insulin stimulates LRP1 trafficking from perinuclear vesicles to the plasma membrane in adipocytes [15]. A similar mechanism for LRP1 translocation in the liver, facilitating the hepatic clearance of atherogenic TRL remnants, was described by Laatsch et al. [14]. Furthermore, we found that this process is dependent on the distal NPxY motif in the LRP1-intracellular domain (LRP1-ICD) [16]. However, the mechanisms regulating perinuclear retention as well as insulin-mediated translocation of LRP1 have not been identified. The protein phosphotyrosine interacting domain containing 1 (PID1) was recently identified by pulldown experiments [17] and *Yeast two Hybrid* [18] as an intracellular binding partner of LRP1. PID1 is a close relative of the autosomal recessive hypercholesterolemia (ARH) protein, an essential hepatic adaptor protein regulating LDLR-mediated endocytosis [19]. ARH can also bind to the distal NPxY motif of LRP1 but it does not regulate endocytic LRP1 function in the liver [20]. By using *in vitro* and *in vivo* genetic models, we here show that PID1 serves as an intracellular retention adaptor protein for LRP1 in hepatocytes. Phosphorylation of the distal NPxY motif disrupts PID1 binding to the LRP1-ICD, which leads to the insulin-dependent translocation and accumulation of LRP1 at the hepatocyte cell surface. Thus, by controlling LRP1 localization, PID1 determines hepatic clearance and consequently plasma levels of pro-atherogenic TRL remnants in the postprandial phase.

## 2 Materials and methods

### 2.1 Generation of knockout mice

**Figure S1:**
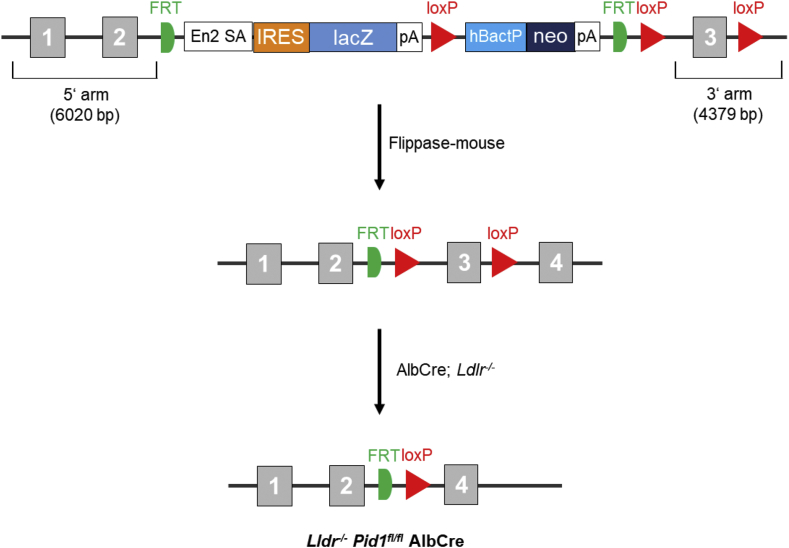
**Generation of *Pid1* knockout mice**. The “Knockout-First-Reporter Tagged Insertion“ *Pid1*^tm1a(KOMP)Wtsi^ allele present in the ESC clone EPD0579_4_G03 and obtained from the NCRR-NIH supported KOMP Repository (www.komp.org) was generated through insertion of the L1L2_Bact_P cassette into intron 2 of the *Pid1* gene at position -84038956 of mouse chromosome 1. Liver-specific *Pid1* knockout mice were generated by crossing “knockout-first” mice with mice expressing flippase [45], yielding mice with floxed 3rd exon of *Pid1*. Crossing these mice with mice expressing liver-specific Cre-recombinase (albumin-promotor driven Cre expression) was used to produce liver-specific *Pid1* knockout mice. By crossing these mice with LDLR deficient mice, double knockout mice (*Ldlr*^*−/−*^*Pid1*^*fl/fl*^ AlbCre+) and respective controls (*Ldlr*^*−/−*^*Pid1*^*fl/fl*^ AlbCre-) were generated.

The “Knockout-First-Reporter Tagged Insertion” Pid1^tm1a(KOMP)Wtsi^ allele present in the ESC clone EPD0579_4_G03 obtained from the NCRR-NIH supported KOMP Repository (www.komp.org) was generated through insertion of the L1L2_Bact_P cassette into intron 2 of the *Pid1* gene at position -84038956 of mouse chromosome 1 (Figure S1). Cells from the clone EPD0579_4_G03 were injected into C57BL/6J blastocysts to generate chimeras. Male chimeric offspring was crossed with C57BL/6J females and the resulting offspring was analysed for transmission of the targeted allele. Pid1^tm1a^ mice were genotyped by PCR using *Pid1*-specific primers flanking the 3′ loxP site downstream of the critical exon 3 (forward 5′-GACATTGAAACCTGCTGCTG-3’; reverse 5′-TCTAGCGCGCTGTTAGTTGT-3′) amplifying PCR products of 405 and 462 bp from the wild-type and targeted locus, respectively. *Pid1*^*tm1a*^ mice were backcrossed to C57BL6/J for at least 7 generations and homozygous mice displaying total PID1 knockout (*Pid1*^*−/−*^) were generated by heterozygous breeding. Wild type littermates were used as controls. To generate tissue-specific PID1-deficient mice, Pid1^tm1a^ mice were crossed with flippase (Flp)-expressing mice to delete the FRT-flanked selection cassette and generate mice with a floxed exon 3 in the *Pid1* gene (Figure S1). These mice were then crossed with mice expressing Cre-recombinase under control of the albumin promotor (Alb-Cre) to generate liver specific knockouts (*Pid1*^fl/fl^-AlbCre+). These mice were subsequently crossed with global LDLR knockout mice to generate liver specific *Pid1*-knockouts (*Ldlr*^−/-^ *Pid1*^fl/fl^-AlbCre+) and respective Cre-negative littermates (*Ldlr*^−/-^ *Pid1*^fl/fl^-AlbCre-) as controls. Animal care and experimental procedures were performed with approval from the animal care committees of the University Medical Center Hamburg-Eppendorf. We used 12–18 week old male mice that were kept on a 12-h light/dark cycle and fed either chow diet or a pro-atherogenic western-type diet (Sniff EF R/M acc.TD88137 mod) for 8 weeks starting at 8 weeks of age. Mutant LRP1-knockin mice lacking a function distal NPxY motif were kindly provided by A. Roebroeck [24].

### 2.2 Cell culture

Human hepatoma cells (HuH7) were transiently transfected with LRP1eGFP and PID1-RFP using FuGene according to the instructions of the company (Promega). Respective vectors were made in-house using standard molecular biology technologies. HuH7 cells were grown in DMEM supplemented with 10% FCS and penicillin/streptomycin/puromycin at 37 °C and 5% CO_2_. Primary murine hepatocytes were prepared by liver perfusion, EDTA dissociation and centrifugation on a self-generating Percoll gradient to separate hepatocytes from non-parenchymal cells [14] and were seeded in DMEM containing 10% FCS to a density of 2 × 10^5^ cells/ml in collagen-coated wells on glass coverslips for immunofluorescence analysis. For PID1 knockdown in primary hepatocytes, the MISSION^®^ esiRNA targeting mouse Pid1 (Sigma–Aldrich, EMU030571) was transfected 4 h after seeding using lipofectamin2000 (Invitrogen by *life technologies*). Experiments were carried out two days after transfection of esiRNA.

### 2.3 Antibodies

Antibodies used in the study were: sheep polyclonal anti-LRP1 “dolly” (made in-house, WB 1:500), mouse monoclonal anti-LRP1 (11H4, WB 1:1200), rabbit polyclonal anti-Flag (Sigma–Aldrich, F7425, WB 1:300), mouse monoclonal *ezview red* anti-Flag M2 affinity gel (Sigma–Aldrich, F2426, 40 μl for IP), mouse monoclonal phosphotyrosine (clone 4G10, Upstate, 05-321, WB 1:1000). Mouse monoclonal anti-β-actin (Sigma–Aldrich, A5441, WB 1:20000), rabbit polyclonal anti-early endosome antigen 1 (EEA1, Abcam, ab50313, IF 1:250); rabbit polyclonal anti-PID1 (Sigma–Aldrich, HPA36103; WB/IF 1:200), rabbit monoclonal anti-LRP1 (Abcam, ab92544, WB 1:10000; IF: 1:500), anti-SR-B1 (kindly provided by F. Rinninger, Hamburg, Germany; WB 1:1000), anti-apolipoprotein E (APOE, Acris, TA326636; 1:1000), goat polyclonal anti-LDLR (R&D Systems, AF2255, WB 1:1000) and rabbit-polyclonal anti-Calnexin (Abcam, ab22595; WB 1:1000). An affinity purified rabbit anti-PID1 antibody was raised against the synthetic peptide LCTTTPLMKARTHSG corresponding to 15 amino acids located in exon 2 of PID1 and used to detect Flag-PID1 in the pull down experiments. All of the following secondary antibodies were purchased from Jackson Immuno Research: donkey anti-mouse Alexa Fluor 555 (IF 1:1000), donkey anti-mouse Alexa Fluor 647 (IF 1:1000), donkey anti-mouse Cy3 (IF 1:500), goat anti-rabbit HRP (WB 1:5000), goat anti-mouse Alexa Fluor 488 (IF 1:1000), goat anti-mouse HRP (WB 1:5000), donkey anti-sheep HRP (WB 1:10000) and donkey anti-rabbit Cy2 (IF 1:1000).

### 2.4 Immunofluorescence

For immunofluorescence experiments, cells were washed with PBS, fixed with 4%PFA and indirect immunofluorescence against LRP1, PID1, and EEA1 was performed using standard procedures. Localization of proteins within the cells was visualized by confocal laser scanning microscopy using a Zeiss LSM710 or a Nikon A1R. To analyse the localization of LRP1 in hepatocytes before and after insulin stimulation, primary wild type and *Pid1*^*−/−*^ hepatocytes were generated as described above and seeded to a density of 1 × 10^5^ cells/ml onto glass coverslips. Hepatocytes were washed once with PBS and incubated with 10 nM Insulin in DMEM supplemented with 10% FCS for 10 min at 37 °C. Then, cells were washed with PBS, fixed with 4% PFA and analysed by using indirect immunofluorescence as described above.

### 2.5 Protein extraction, SDS-PAGE and western blotting

Western blots were performed using standard procedures. Cells were lysed in lysis buffer (2 mM CaCl_2_, 80 mM NaCl, 1% TritonX-100, 50 mM Tris/HCl, pH 8.0) supplemented with Complete Mini protease inhibitor cocktail (Roche). Snap-frozen tissue samples were lysed in RIPA buffer (50 mM Tris-HCl pH7.4, 5 mM EDTA, 150 mM NaCl, 1 mM Na-Pyrophosphate, 1 mM NaF, 1 mM Na-Vanadate, 1% NP-40) supplemented with Complete Mini protease inhibitor cocktail (Roche) and 0.1% SDS using the TissueLyser (Qiagen). Protein concentrations were determined using the Lowry method and subsequently, proteins were separated on NuPAGE Bis-Tris 4–12% gradient gels (Invitrogen) or 10% Tris-glycine gels. For western blotting, proteins were transferred to nitrocellulose membranes, blocked for 1h in 5% milk in TBS-T (20 mM Tris, 150 mM NaCl, 0.1% (v/v) Tween 20) and incubated overnight at 4 °C in the respective primary antibodies, diluted in 5% BSA in TBS-T. After washing in TBS-T, the membranes were incubated with respective secondary horseradish peroxidase-labelled antibodies. Signals were detected with enhanced chemiluminescence (ECL) using Amersham Hyperfilm (GE Healthcare) or Amersham Imager 600 (GE), and quantification was carried out using ImageJ.

### 2.6 GST-pulldown experiments

**Figure S2:**
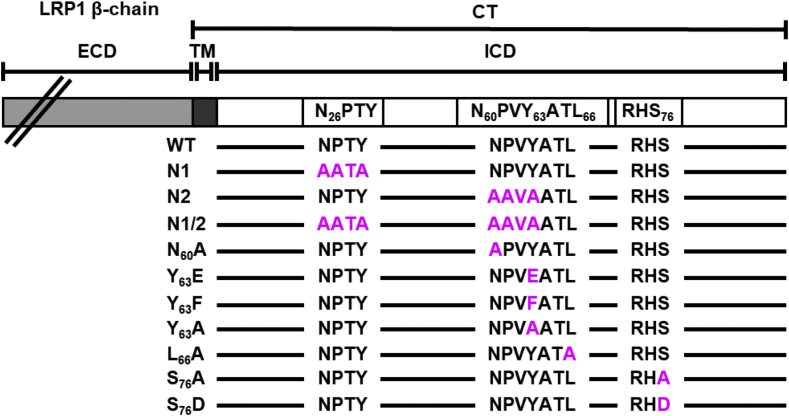
**Domain structure and potential PID1 binding motifs of LRP1**. The extracellular (ECD), as well as the transmembrane (TM) and intracellular (ICD) domains forming the carboxy-terminus (CT) are indicated. The proximal and distal NPxY motifs as well as the RHS motif of the ICD are indicated. Wild type (WT) and mutant LRP1-ICD lacking the functional proximal (N1), distal (N2) or both (N1/2) NPxY domains as well as amino acid substitutions within these domains created for biochemical binding analyses are highlighted in pink.

For purification of GST-fusion proteins, the GST expression vector (pGex-6P, GE Healthcare) as well as the Flag-expression vector pCMV-Tag2B, both containing the respective insert (murine *PID1*; wild type and mutated *LRP1* ICD; see Figure S2 for structure and mutations of the intracellular LRP1 domain) were transformed into E. coli BL21 (Stratagene). GST fusion proteins were purified via Glutathion Sepharose 4B (GE Healthcare). LRP1-ICDs were released from the column by incubation with PreScission protease (GE Healthcare). Integrity of all recombinant proteins was confirmed by SDS-PAGE. For pulldown analysis either cell or tissue lysates (150 μg) were incubated over night at 4 °C with GST-fusion proteins. Subsequently pulldown and unbound fractions were separated by centrifugation for 3 min at 9000 rpm. Supernatants were harvested (unbound fraction) and sepharose pellets were washed six times using PBS. Finally, pellets were reduced, and PID1-interacting proteins were analysed by Western blotting.

### 2.7 Immunoprecipitation

To analyse LRP1-PID1 interaction, constructs coding for the Flag-PID1 fusion protein and the LRP1-cytoplasmic tail were co-expressed in human embryonic kidney cells (HEK 293T). Cell lysates were incubated with anti-Flag M2 affinity gel or Protein G bound LRP1 specific antibody for 12 h at 4 °C on a rotator, respectively. Elution was performed using NuPage SDS sample buffer containing reducing agent (1x, Invitrogen by life technologies) for 5 min at 95 °C. Proteins were detected by Western blotting.

### 2.8 mRNA expression analysis

Total RNA was isolated from cells and organs using TRIzol (Invitrogen by *life technologies*) and a NucleoSpin RNAII kit (Macherey und Nagel) according to manufacturer's instructions. After DNase treatment (RNase-free DNase set, Qiagen), 1 μg of RNA was used for cDNA preparation (High-Capacity cDNA Reverse Transcription Kit, Applied Biosystems) according to manufacturer's instructions. Quantitative real-time RT-PCR was performed using assays-on-demand primer/probe sets provided by Applied Biosystems (Assay IDs: mPid1, Mm01545237_m1; mLrp1, Mm00464608_m1; mTbp, Mm00446973_m1). Relative mRNA expression was calculated by the ΔΔCT method and normalized to housekeeper mRNA (*Tbp*) as described [21].

### 2.9 Biotinylation

*In vivo* biotinylation was performed as described in detail elsewhere [22]. Briefly, wildtype and *Pid1*^*−/−*^ mice were i.p. injected with acepromazin (Sigma, 3 mg/kg body weight) to promote vasodilatation and anaesthetized using ketamine/rompun. Then, mice were perfused first with 10 ml of pre-warmed PBS supplemented with 10% dextrane 40 for 10 min, subsequently with biotinylation buffer containing non-permeable, cleavable 1 mg/ml EZ Link Sulfo-NHS-SS-Biotin (Thermo Scientific) in PBS containing 10% dextrane 40 for 10 min at 37 °C, and finally with 50 mM Tris/HCl in PBS containing 10% dextrane 40 for 10 min at 37 °C to inactivate EZ Link Sulfo-NHS-SS-Biotin. Livers were lysed in cell lysis buffer and equal protein amounts of each sample were incubated with pre-washed PierceTM Streptavidin Magnetic Beads (Thermo Scientific) according to manufacturer's instructions at 4 °C for 90 min on a rotator. Afterwards, magnetic beads were washed twice with TBS containing Tween20 and twice with PBS. Biotinylated proteins were subsequently eluted with NuPage SDS sample buffer containing reducing buffer (1x, Invitrogen by life technologies) and analysed by SDS-PAGE and western blotting.

### 2.10 Plasma analysis

Mice were fasted for 4 h. Blood was collected via the tail vein or by cardiac puncture of anaesthetized mice. Plasma cholesterol and triglyceride were determined using commercial kits (Roche) according to manufacturer's instructions. For analysis of lipoprotein classes, fast protein liquid chromatography (FPLC) was performed. Plasma samples from several mice were pooled and size exclusion chromatography was performed on a S6-FPLC column. Cholesterol- and triglyceride content was determined in the different fractions.

### 2.11 Lipid uptake studies

Oral fat tolerance tests traced with 12 KBq [9,10-^3^H(N)]-triolein/g body weight were performed as described [23]. Briefly, mice were fasted for 4 h before receiving an oral gavage of olive oil containing [9,10-^3^H(N)]-triolein. Four hours after gavage, mice were anaesthetized with ketamine/rompun. Then, mice were transcardially perfused with PBS containing 10U/ml heparin to release LPL-bound TRLs. Organs were solubilized in 10x (v/w) solvable (Perkin Elmer) at 60 °C and radioactivities were determined using Aquasafe 300 Plus scintillation liquid (Zinsser Analytic) in a liquid scintillation counter (Perkin Elmer Tricarb).

### 2.12 Intravital microscopy

Intravital microscopy was performed as described previously [24]. Briefly, fasted mice were i.p. injected with saline or insulin-saline (1U/kg body weight). After 10 min, mice were anaesthetized using isoflurane inhalation anaesthesia. The tail vein was catheterized, skin and peritoneum were opened, and the liver was prepared and attached to a cover glass. Microscopy was performed using a Nikon A1 confocal microscope equipped with a Resonant Scanner for image acquisition at 30 fps. For visualization of LRP1 translocation, 100 μg of Cy5-labelled anti-LRP1 antibody in 100 μl saline were injected via tail vein catheter and the binding of the antibody was monitored for 10 min. Antibody labelling was performed using a commercial kit (Cy5-labelling kit GE Healthcare). 1 mg of antibody was incubated with the dye mix in a 0.1 M Bicarbonate-Solution for 1 h at room temperature and 300 rpm shaking. Afterwards, free dye was removed using a PD10 size exclusion column (GE healthcare). During this procedure, the buffer was replaced by 0.9% NaCl solution.

### 2.13 Determination of hepatic lipid content

To measure the hepatic lipid content, tissue samples were homogenized in RIPA buffer (see above) and protein concentration was determined using the Lowry method. Triglyceride content in the liver extract was measured with commercial triglyceride kit (Roche) according to manufacturer's instructions. For quantification of cholesterol content, 50 μl of liver homogenate were mixed with 1 ml chloroform/methanol (8:5 v/v) and centrifuged at 13000 rpm for 5 min. The supernatant was dried and reconstituted in 200 μl AmplexRed reaction buffer (Invitrogen) for 20 min at 50 °C on a shaker. Cholesterol concentration was measured using AmplexRed Cholesterol Assay Kit (Invitrogen) according to the manufacturer's instructions.

### 2.14 Statistical analyses and data processing

Two-tailed, unpaired Student's t-test was performed to calculate statistical significance. P < 0.05 was considered to be statistically significant.

## 3 Results

### 3.1 Interaction of PID1 and LRP1

**Figure 1:**
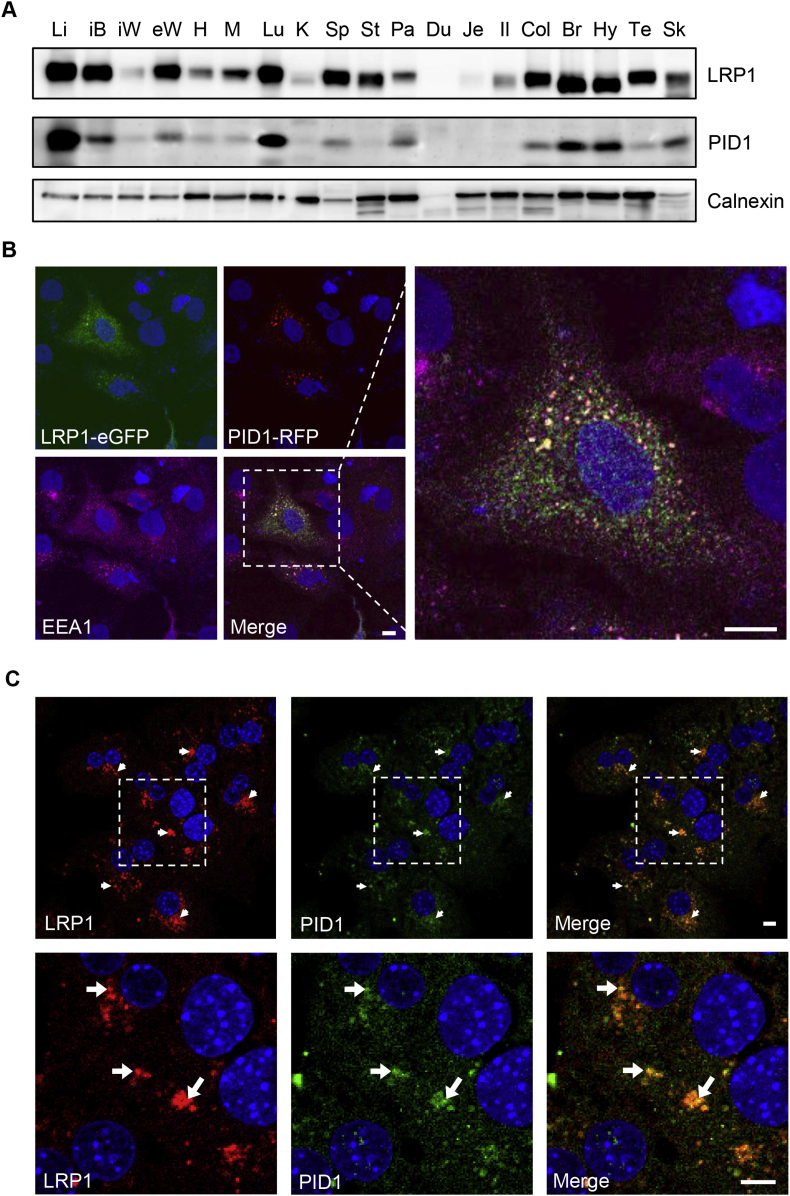
Expression and intracellular localization of PID1. (A) Protein expression of LRP1 and PID1 by western blot analysis in different organs of a C57BL6/J mouse. Calnexin was used as loading control. Li - liver; iB - interscapular brown adipose tissue; iW - inguinal white adipose tissue; eW - epididymal white adipose tissue; H – heart; M− quadriceps muscle; Lu – lung; K – kidney; Sp – spleen; St – stomach; Pa – pancreas; Du – duodenum; Je – jejunum; Il – ileum; Col – colon; Br – whole brain; Hy – hypothalamus; Te – testis; Sk – skin. (B) Human HuH7 hepatoma cells were transfected with expression vectors coding for PID1-RFP (red) and LRP1-eGFP (green). EEA1 (purple) was detected by indirect immunofluorescence and confocal images were taken. A magnification of the merged image is shown on the right picture (bars: 10 μm). Nuclei were stained using DAPI (blue). (C) Indirect immunofluorescence was performed to visualize endogenously expressed LRP1 (red) and PID1 (green) in murine wild type primary hepatocytes. Arrows indicate co-localization. Nuclei were stained using DAPI (blue). Bars: 5 μm.

**Figure 2:**
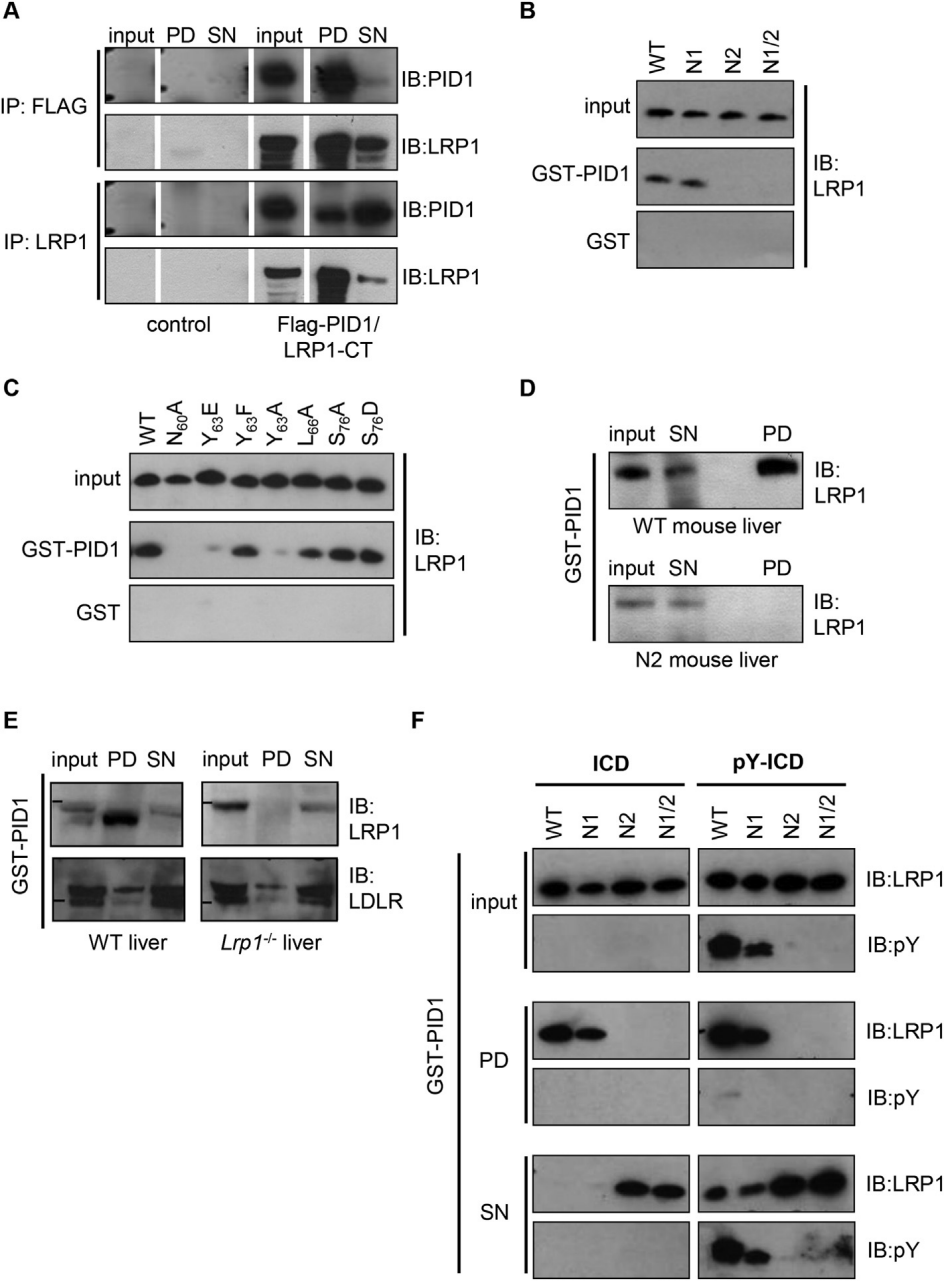
Interaction of PID1with the distal NPxY-motif of LRP1. (A) Co-immunoprecipitation (IP) with lysates of non-transfected control cells and HEK293T cells co-transfected with Flag-PID1 and the C-terminal domain of LRP1 (LRP1-CT). IP was performed using an anti-Flag or an anti-LRP1 antibody. After western blotting, PID1 or LRP1 were detected using specific antibodies in the IP fractions (I: input; SN: supernatant). (B) GST-PID1 pulldown experiments with wild type (WT) and mutant LRP1-ICD lacking the functional proximal (N1), distal (N2) or both (N1/2) NPxY domains of LRP1-ICD. (C) GST-PID1 pulldown experiments with WT and LRP1-ICD mutated in single amino acids within the distal NPxY motif. (D) GST-PID1 pulldown studies with liver lysates isolated from WT and LRP1 knockin mice lacking a functional distal NPxY domain. (E) GST-PID1 pulldown studies with liver lysates isolated from WT and liver-specific *Lrp1*^−/−^ mice. (F) GST-PID1 pulldown with non-phosphorylated LRP1-ICD (ICD) and v-Src kinase phosphorylated LRP1 (pY-ICD).

We determined the expression PID1 and LRP1 in a mouse organ panel to identify tissues with high abundance of both these proteins as potential sites of interaction (Figure 1A). LRP1 is widely expressed with the highest levels in the liver, followed by brown adipose tissue, lung, and brain. Notably, the pattern of PID1 is very similar with the highest expression also in the liver (Figure 1A). This co-expression is consistent with a potential role of PID1 as an adaptor protein controlling LRP1 function in liver and other organs. To investigate the interaction of the two proteins at the cellular level, human hepatoma HuH7 cells were transfected with PID1-RFP and LRP1-eGFP. Confocal microscopy indicated that these fluorescent-tagged versions of LRP1 and PID1 co-localized in EEA1-positive early endosomes primarily located within the perinuclear region (Figure 1B). A similar distribution was found for endogenous LRP1 and PID1 in primary murine hepatocytes (Figure 1C), providing evidence for an interaction of the two proteins. To determine the molecular basis of this interaction, immunoprecipitation (IP) and GST-based pulldown experiments were conducted. Co-transfection studies in HEK293T cells showed binding of FLAG-tagged PID1 to the LRP1 carboxyl-terminus (LRP1-CT), which contains the cytoplasmic as well as the transmembrane domain of the LRP1-β-chain (Figure 2A and Fig. S2). The LRP1-ICD contains three putative phosphorylation motifs, a serine phosphorylation motif (RxS_76_), and two tyrosine phosphorylation sites within the proximal and distal NPxY-motifs. The latter one overlaps with a YxxL motif, forming a ^60^NPxYxxL^66^ double motif (Fig. S2). The potential role of these motifs for the interaction of PID1 and LRP1 was further characterized using pulldown experiments with a GST-PID1-fusion protein in combination with recombinant wild type (WT) and mutant LRP1-ICDs (Figure 2B). GST-PID1 was able to bind wild type LRP1-ICD as well as the LRP1-ICD lacking a functional proximal NPxY domain. However, the GST-PID1 did not interact with LRP1-ICDs lacking a functional distal NPxY domain (Figure 2B), indicating that PID1 binds to the distal NPxY motif. Single amino acid substitution identified the asparagine at position 60 and the tyrosine at position 63 within the distal NPxY motif as being critical for the LRP1-PID1 interaction (Figure 2C). *In vivo* experiments confirmed these findings, as purified GST-PID1 fusion protein was able to pull down LRP1 (Figure 2D, upper panel) but not the LDLR (Figure 2E) from wild type liver lysates. In line with the *in vitro* data and as shown in the lower panel of Figure 2D, GST-PID1 was unable to pull down LRP1 from liver lysates of knockin mice expressing a mutated distal NPxY motif [25].

It is well established that phosphorylation of NPxY motifs determines binding affinity of adaptor proteins [26]. Thus, we investigated whether phosphorylation of the tyrosine residue in the distal NPxY of LRP1 affects PID1 binding in pulldown experiments with LRP1-ICD constructs mutated at tyrosine 63. The residue was mutated to glutamate (Y_63_E) and to phenylalanine (Y_63_F) to mimic a constitutively phosphorylated and non-phosphorylated distal NPxY, respectively. The Y_63_F but not the Y_63_E protein was pulled down (Figure 2C), indicating that PID1 binds only to the non-phosphorylated LRP1-ICD. The observed sensitivity of PID1 binding to phosphorylation of the LRP1 NPxY motif was confirmed using constructs that were partially *in vitro* phosphorylated by v-Src-kinase [27]. In the pull down fraction, we detected only non-phosphorylated LRP1-ICD, as indicated by absence of phospho-tyrosine (pY) immunoreactivity (Figure 2F). In contrast, both phosphorylated wild type and mutated proteins lacking only the functional proximal NPxY were detected in the supernatant only after treatment with v-Src kinase, coinciding with their phosphorylation (Figure 2F). Constructs lacking the distal NPxY motif did not get phosphorylated by v-Src-kinase treatment, and as shown before did not bind PID1 as the respective LRP1-ICDs were found in the supernatants. Altogether, these results demonstrate that PID1 co-localizes with LRP1 in perinuclear endosomes of hepatocytes and binds specifically to the non-phosphorylated distal NPxY motif of LRP1.

### 3.2 PID1 controls LRP1 localization and abundance in hepatocytes *in vitro* and *in vivo*

**Figure 3:**
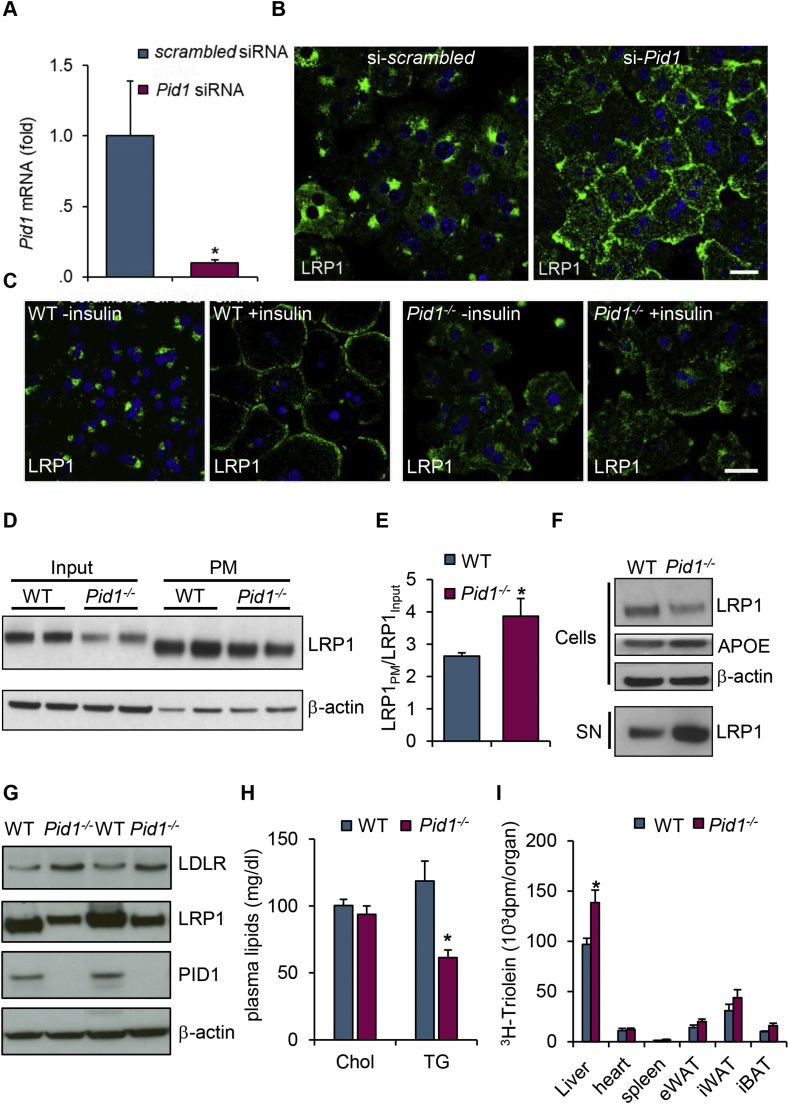
Effects of PID1 deficiency on LRP1 expression, localization and function. (A) Expression of *Pid1* mRNA and (B) localization of endogenous LRP1 (green) in primary murine hepatocytes after transfection with scrambled or *Pid1*-specific siRNA. Nuclei are stained using DAPI (blue). Bar: 20 μm. (C) Primary hepatocytes isolated from WT and *Pid1*^−/−^ mice were treated with or without 10 nM insulin for 15 min prior to indirect immunofluorescence against LRP1 (green). Nuclei are stained with DAPI (blue). Bar: 20 μm. (D) *In vivo* cell surface biotinylation was performed in WT and *Pid1*^−/−^ mice. LRP1 expression was quantified in total liver lysates (Input) and the isolated plasma membrane (PM) fraction. β-actin was used as loading control. (E) Densitometric analysis of the western blots comparing the ratio of plasma membrane to total LRP1 protein (n = 4). (F) LRP1 detection in cell lysates and supernatants (SN) of primary hepatocytes isolated from WT and *Pid1*^−/−^ mice. (G) Western blot analysis of PID1, LRP1, LDLR and β-actin in liver lysates of WT and *Pid1*^−/−^ mice. (H) Plasma cholesterol and triglycerides in WT and *Pid1*^−/−^ mice (n = 7–8). (I) Organ-specific uptake of ^3^H-labelled triolein after oral fat gavage in WT and *Pid1*^−/−^ mice (n = 7–8). Data are presented as mean values ± s.e.m. *P < 0.05.

**Figure S3:**
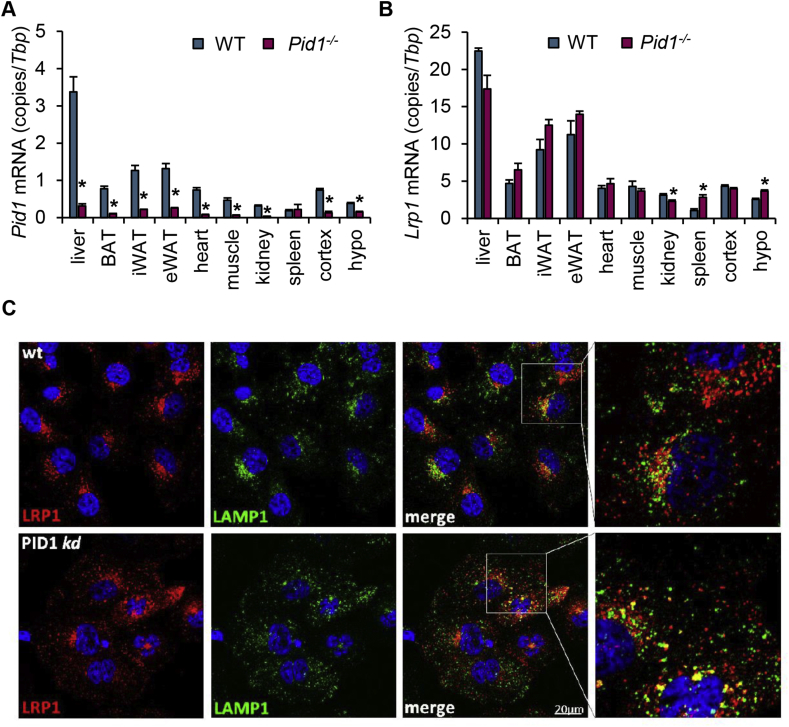
**mRNA expression of PID1 and LRP1 in wild type and *Pid1***^***−/−***^**mice**. (A) *Pid1* and (B) *Lrp1* mRNA expression levels in different organs of WT and *Pid1*^−/−^ mice. Copy numbers were normalized on the expression of the housekeeping gene TATA-box-binding-protein *Tbp*. (BAT, brown adipose tissue; iWAT, inguinal white adipose tissue; eWAT, epididymal white adipose tissue; hypo., hypothalamus). Mean values ± s.e.m. with n ≥ 5 are shown. *P < 0.05. (C) Human HuH7 hepatoma cells were transduced with lentiviruses expressing either shRNA targeting human PID1 (PID1 *kd*) or a scrambled control shRNA (wt). Indirect immunofluorescence was performed to visualize endogenously expressed LRP1 (red) and LAMP1 (green). Nuclei were stained using DAPI (blue). Bar: 20 μm.

To investigate the role of PID1 for LRP1 function, we performed siRNA-mediated silencing of PID1 in primary murine hepatocytes (Figure 3A). Typical LRP1 perinuclear staining was observed in control cells, whereas PID1 knockdown resulted in a predominant plasma membrane localization of LRP1 (Figure 3B). These results indicate that PID1 is crucial for LRP1 localization. Moreover, its depletion cannot be compensated by other hepatic adaptor proteins, which prompted us to study the *in vivo* relevance of PID1 in total body knockout (*Pid1*^*−/−*^) mice. The *Pid1*^*−/−*^ mice were viable and showed an effective reduction of *Pid1* mRNA in various organs (Figure S3A) with minor effects on LRP1 expression (Figure S3B). Hepatocyte LRP1 has been shown to translocate from perinuclear regions to the plasma membrane in response to insulin treatment [14]. This insulin-dependent LRP1 localization was confirmed in primary hepatocytes isolated from wild type mice (Figure 3C). Similar to the results obtained with siRNA-mediated PID1 knockdown (Figure 3B), we observed high abundance of LRP1 at the cell surface already under basal conditions in primary hepatocytes isolated from *Pid1*^*−/−*^ mice (Figure 3C). Notably, this distribution was not further altered by insulin administration (Figure 3C). The altered LRP1 localization was confirmed by *in vivo* cell surface biotinylation experiments (Figure 3D,E), as an enrichment of plasma membrane LRP1 over whole tissue was observed in the absence of PID1. However, total and cell surface LRP1 levels were lower in livers of *Pid1*^−/−^ compared to wild type mice. In line with the *in vivo* data, total LRP1 protein was also decreased in *Pid1*^−/−^ primary hepatocytes (Figure 3F). In conjunction, we observed markedly increased LRP1 content in cell culture supernatants of Pid1^−/−^ primary hepatocytes (Figure 3F), indicating that the reduction is explained by accelerated shedding of the receptor. Additionally, altered intracellular sorting of LRP1 leading to lysosomal degradation may contribute to the reduced total LRP1 levels. In line with this, we observed partial co-localization with the lysosomal marker LAMP1 in PID1-knockdown hepatoma cells (Figure S3C). We also found a profound increase in hepatic LDLR expression in *Pid1*^*−/−*^ mice (Figure 3G). The latter observation is in line with the previously reported up-regulation of the LDLR in liver-specific LRP1 knockout mice [8]. Consistently, plasma triglycerides were significantly decreased in *Pid1*^*−/−*^ mice compared to wild type controls (Figure 3H). This reduction in plasma triglycerides is explained by increased TRL uptake into livers of *Pid1*^*−/−*^ mice as shown by an *in vivo* metabolic clearance study using radioactive ^3^H-triolein (Figure 3I). Thus, PID1 inactivation leads to a reduction of hepatic LRP1 protein that results in a compensatory LDLR up-regulation and an associated accelerated lipoprotein clearance.

### 3.3 PID1 is an LRP1 perinuclear endosome retention adaptor protein

**Figure 4:**
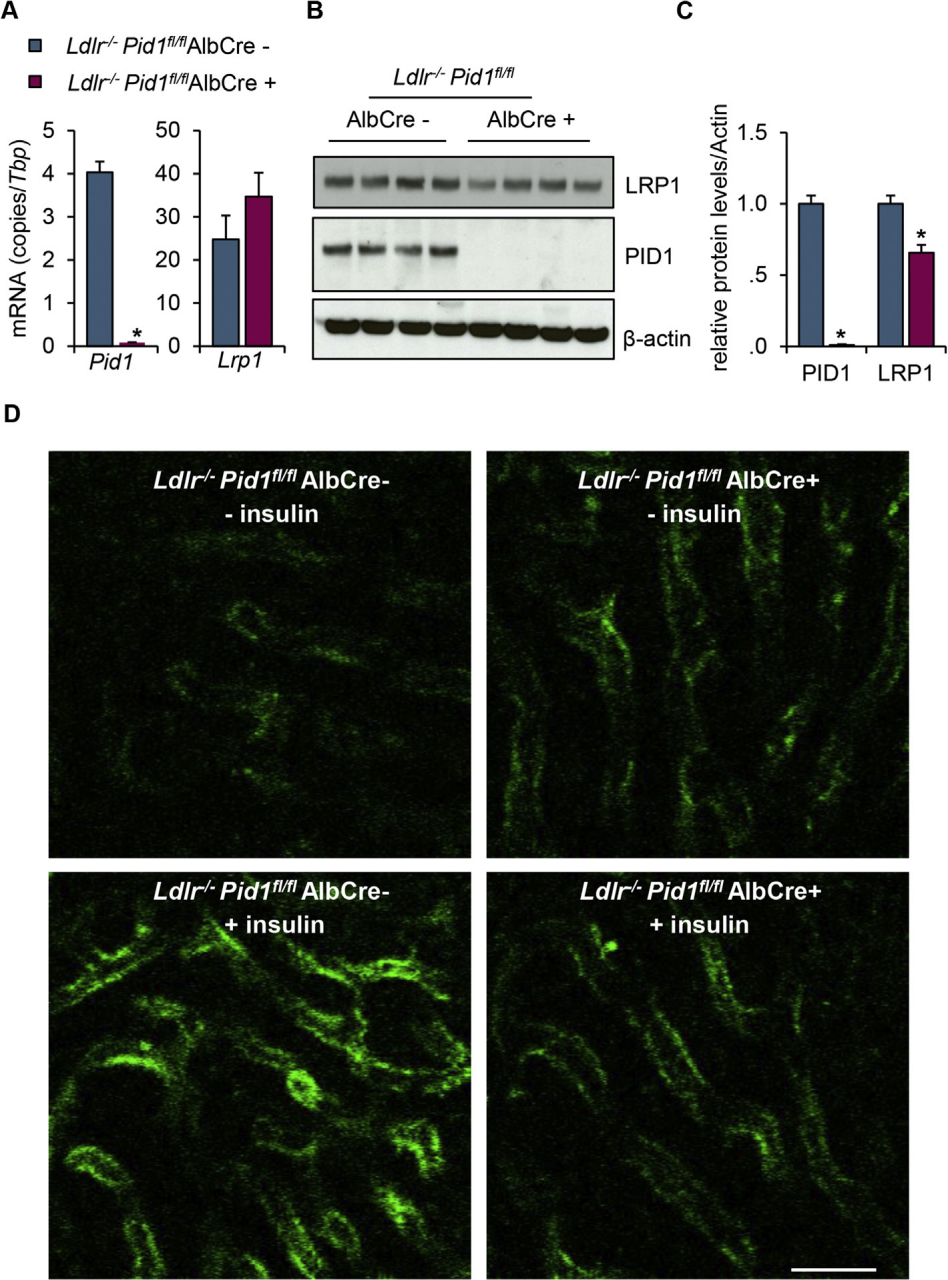
Expression and insulin-dependent localization of LRP1 in liver-specific *Pid1*^−/−^ mice knockout mice lacking the LDLR. To generate LDLR deficient mice with (*Ldlr*^*−/−*^*Pid1*^fl/fl^ AlbCre-) or without (*Ldlr*^*−/−*^*Pid1*^fl/fl^ AlbCre+) hepatic PID1, LDLR deficient mice were crossed with Pid1-floxed mice and mice expressing Cre recombinase driven by the liver-specific albumin promotor. (A) mRNA and (B) protein expression of PID1 and LRP1 in livers. (C) Densitometric analysis of the Western blots shown in B (n = 4). (D) Intravital microscopy of livers after treatment with either saline (- insulin) or 1 U/kg insulin (+insulin). Cell surface LRP1 was visualized by injection of a Cy5-labelled antibody against LRP1 (green). Intravital microscopy was performed over a period of 10 min (see Supplemental Video): Images shown here were taken 10 min after antibody injection. Bars 25 μm.

We generated liver-specific PID1-knockout mice on a LDLR-deficient background to separate direct effects of hepatic PID1 depletion on LRP1 localization and abundance independent from its indirect effects on hepatic LDLR expression (see Figure 3G). For this purpose, floxed *Pid1* (*Pid1*^*fl/fl*^) mice (Figure S1) were crossed with mice expressing Cre-recombinase exclusively in the liver via the albumin promotor (AlbCre+), and subsequently crossed with *Ldlr*^−/-^ mice. Compared to controls (*Ldlr Pid1*AlbCre-), recombination led to an effective reduction of *Pid1* mRNA expression in livers of *Ldlr*^*−/−*^ *Pid1*^*fl/fl*^AlbCre+, whereas *Lrp1* mRNA levels were unaltered (Figure 4A). Western blot analysis confirmed effective PID1 knockout, which, similar to the total PID1 knockout mice, was associated with a decrease in total hepatic LRP1 protein abundance (Figure 4B). To study the impact of PID1 on LRP1 redistribution from perinuclear compartments to the plasma membrane *in vivo*, we performed intravital microscopy in *Ldlr Pid1*AlbCre- and *Ldlr Pid1*^*fl/fl*^AlbCre + mice. Next we injected mice with insulin or saline followed by a fluorescently labelled antibody against the extracellular domain of LRP1 to visualize LRP1 abundance at the plasma membrane as well as insulin-dependent translocation. In *Ldlr Pid1*AlbCre-controls, we observed a low basal abundance of LRP1 at the cell surface of hepatocytes (Figure 4D upper left, Supplemental Video). Insulin administration strongly increased cell surface fluorescence (Figure 4D lower left, Supplemental Video), indicating LRP1 translocation to the plasma membrane. In *Ldlr Pid1*AlbCre+, basal LRP1 staining was higher compared to the controls (Figure 4D upper right, Supplemental Video). Similar to the results obtained in PID1-deficient primary hepatocytes (Figure 3C), insulin treatment did not further increase LRP1 plasma membrane levels in the absence of PID1 (Figure 4D lower right, Supplemental Video).

Supplementary video related to this article can be found at https://doi.org/10.1016/j.molmet.2018.07.010.

The following is the supplementary data related to this article:

Multimedia component 1 1 Multimedia component 1

Altogether, these data indicate that PID1 is a retention adaptor protein that keeps LRP1 in perinuclear endosomes, preventing its shedding at the plasma membrane of hepatocytes in the basal state. Moreover, unlike in wild type hepatocytes insulin was unable to stimulate translocation of LRP1 to the plasma membrane in PID1-deficient hepatocytes, suggesting that PID1 controls the endocytic capacity of LRP1 in the postprandial state.

### 3.4 Hepatic PID1 regulates plasma lipoprotein levels

**Figure 5:**
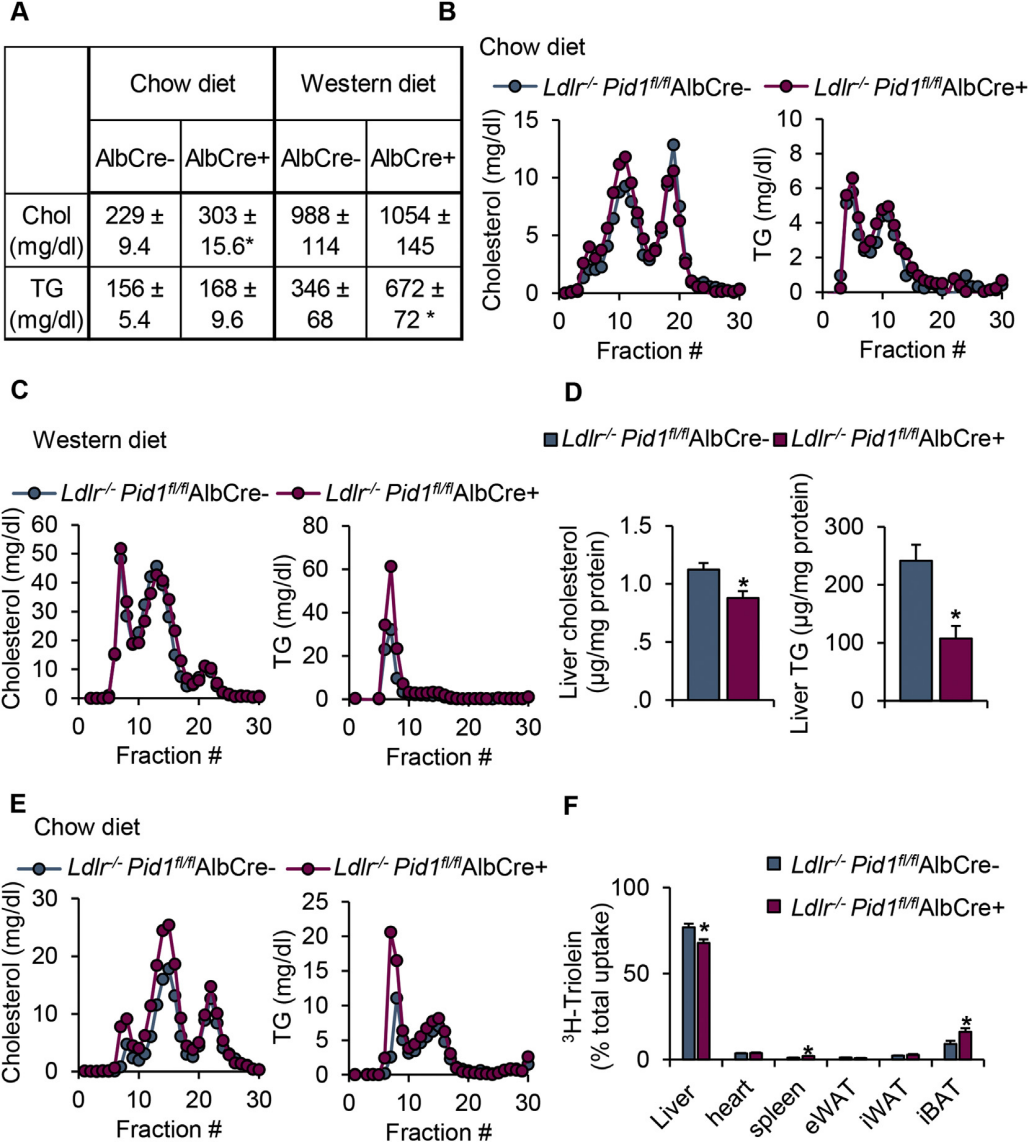
Plasma lipid levels and uptake of postprandial lipoproteins in liver-specific *Pid1*^−/−^ mice knockout mice lacking the LDLR. LDLR deficient mice with (*Ldlr*^*−/−*^*Pid1*^fl/fl^ AlbCre-) or without (*Ldlr*^*−/−*^*Pid1*^fl/fl^ AlbCre+) hepatic PID1 were investigated. (A) Total plasma lipid levels in mice fed a chow (n = 9) or Western type diet (n = 6). (B,C) Lipoprotein profiling of pooled plasma using fast protein liquid chromatography (FPLC) analysis of mice fed a (B) chow or (C) Western type diet. (D) Liver cholesterol and triglyceride levels normalized to protein content in fasted chow-fed mice (n = 4). (E) Lipoprotein profile and (F) organ-specific uptake of ^3^H-labelled triolein after oral fat gavage (n = 6). Data are presented as mean values ± s.e.m. *P < 0.05.

To determine the impact of hepatic PID1 on plasma lipoprotein metabolism on the *Ldlr*^*−/−*^ background, *Ldlr Pid1*AlbCre- and *Ldlr Pid1*AlbCre + mice were fed either a chow or a cholesterol-rich Western-type diet (WTD). Plasma analysis of fasted chow fed mice revealed no significant alterations in plasma triglycerides and a modest 1.3-fold increase in total plasma cholesterol levels (Figure 5A). This increment in circulating cholesterol levels coincided with a slight increase in cholesterol-rich IDL and LDL particles (Figure 5B). Fasted WTD-fed mice presented with a 2–3fold increase in plasma lipid levels compared to the chow-fed group. Under WTD-fed condition the plasma triglycerides levels in the PID1-deficient group were 1.9-fold higher compared to the *Ldlr Pid1*AlbCre-controls, which was reflected by higher triglyceride content in the TRL fraction (Figure 5A,C). Moreover, WTD-fed *Ldlr Pid1*AlbCre + mice had a significant reduction in liver lipid content (Figure 5D). The data are consistent with impaired clearance of postprandial remnant lipoproteins in *Ldlr Pid1*AlbCre + mice. To address this concept we performed an oral fat tolerance study in chow fed mice using olive oil combined with radioactive ^3^H-triolein. Under this postprandial condition, we observed an accumulation of TRL and remnant lipoproteins (Figure 5E), which was accompanied by a reduction of hepatic triolein uptake in *Ldlr Pid1*AlbCre + mice compared to controls (Figure 5F). Altogether, these data indicate that hepatic PID1 is critically involved in the regulation of LRP1 endocytic function, and thereby modulates plasma lipoproteins levels especially in the postprandial state.

## 4 Discussion

**Figure 6:**
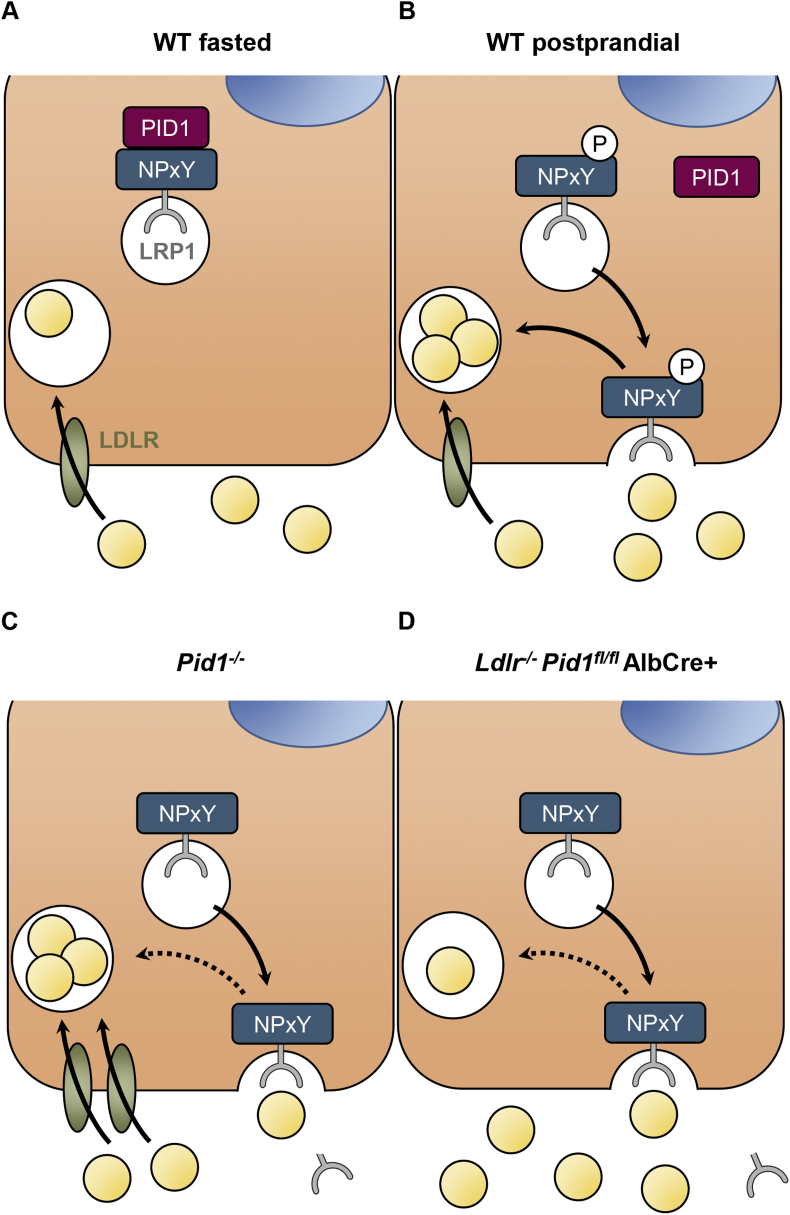
The retention adaptor PID1 as a regulator of LRP1 localization and function. (A) In hepatocytes of fasted wild type mice, the adaptor protein PID1 binds to the distal NPxY motif of the intracellular domain of LRP1. This causes the retention of LRP1 in perinuclear compartments. (B) In the postprandial phase, anabolic signalling mediated by insulin causes the phosphorylation of the distal NPxY motif of LRP1, resulting in the dissociation of PID1. Subsequently, LRP1-containing endosomes vesicles fuse with the plasma membrane leading to exposure of LRP1 to mediate effective endocytosis of postprandial lipoproteins. (C) In PID1-deficient hepatocytes, LRP1 is not retained in perinuclear compartments, and thus distributed to the plasma membrane. The constitutive presence at the cell surface leads to increased shedding and consequently lower LRP1 protein levels. Despite impaired LRP1 function, the compensatory up-regulation of the LDLR causes lower plasma lipoprotein levels. (D) Accordingly, when the LDLR is absent the loss of LRP1 by shedding leads to impaired endocytosis and accumulation of plasma lipoproteins.

**Figure S4:**
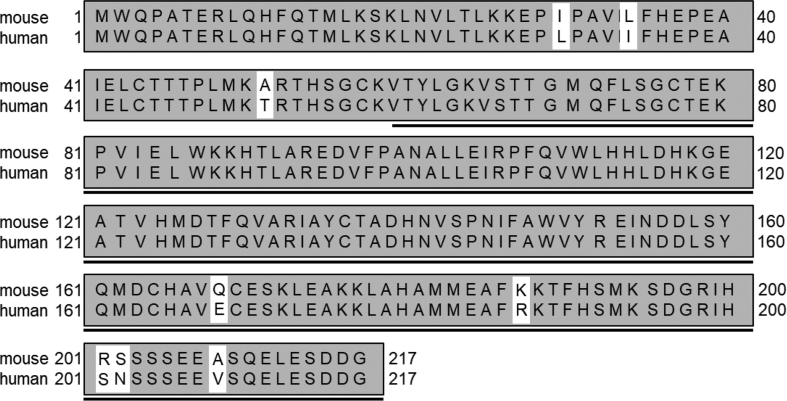
**Sequence alignment of human and murine PID1 proteins**. Variable amino acids are indicated in white and the phospho-tyrosine binding domain is underlined.

PID1 was originally identified in a suppression subtractive hybridization study as a novel gene being expressed to a higher level in subcutaneous adipose tissue from obese subjects [28], which suggests that PID1 could play a role in the development of obesity associated metabolic abnormalities such as hyperlipidemia and hyperglycemia. Sequence homology between murine and human PID1 is very high (96%, Fig. S4), implying a critical role of this adaptor protein for cellular trafficking. In line, PID1 levels were found to be inversely correlated with glucose uptake in adipocytes and myotubes [29], [30]. Mechanistic insights into how PID1 functions *in vivo* are lacking, and the role of PID1 in the liver has not been studied at all. Using proteomic approaches, two studies showed that the cytosolic protein PID1 is an intracellular binding partner for LRP1 [17], [18], a lipoprotein receptor showing a perinuclear distribution in the basal state [14], [31], [32]. In this study, we provide evidence that PID1 via its binding to the non-phosphorylated, distal NPxY motif in the LRP1-ICD, sequesters the receptor in endosomal compartments, thereby controlling LRP1 function. PID1 contains a phospho-tyrosine binding domain but lacks additional motifs that could mediate the association to the endosomal sorting machinery. In contrast, ARH, a close relative of PID1, binds the NPXY motif of the LDLR and thereby attaches this receptor to the endocytic machinery to mediate clathrin-dependent endocytosis [33]. Here we show that the dissociation of PID1 from the distal NPXY motif results in higher abundance of LRP1 at the plasma membrane. Together with the observation that the distal PID1-binding NPXY motif of LRP1 does not serve as an endocytosis signal [34], our data indicate that PID1 does not connect LRP1 to clathrin and/or AP-2 for endocytosis. Rather, we propose that PID1 belongs to a wider class of retention adaptor proteins that retain signalling and endocytic receptors in a perinuclear compartment during quiescent phases. The phosphorylation of such NPxY-containing receptors can be triggered by a signalling event leading to the dissociation of these retention adaptors. It has been demonstrated that LRP1 is phosphorylated in response to insulin [35], [36], and we previously found that insulin mediates the translocation of LRP1 from hepatic perinuclear recycling endosomes to the plasma membrane in the postprandial state [14]. In the present study, we show that phosphorylation of the tyrosine residue within the distal NPxY motif resulted in dissociation of PID1 from the LRP1-ICD. Thus, PID1 functions as an insulin-dependent molecular switch that regulates cellular LRP1 distribution and function. As summarized in the model shown in Figure 6, PID1 binds to non-phosphorylated LRP1-ICD prevalent in the fasted state. Upon postprandial insulin stimulation intracellular tyrosine kinases phosphorylate the distal NPxY motif of LRP1. This event lowers the binding affinity of PID1, enabling interaction with other adaptor proteins that sort LRP1 to the plasma membrane. One of these proteins could be sorting nexin 17 (SNX17), an adaptor that binds to the proximal NPxY motif known to mediate transport of LRP1 from endosomal compartments to the cell surface [37]. Notably, this process requires proper phosphorylation of Y^63^ within the distal NPxY motif [38], [39]. This observation aligns with our model where PID1 is the molecular switch determining LRP1 localization and translocation possibly by preventing SNX17 binding in the basal state but allowing SNX17 binding after insulin-stimulated LRP1 phosphorylation (Figure 6). Potential interaction partners linking PID1 to the perinuclear recycling compartment are e.g. vesicular sorting proteins forming the retromer, a complex that regulates endosome-to-plasma membrane trafficking of various receptors [40]. Alternatively, SNX17 recently has been linked to the endosomal cargo sorting pathway as a retromer-independent sorting pathway involving retriever, a recently discovered complex that couples to the CCC and WASH complexes to prevent lysosomal degradation and promote cell surface recycling [41]. Interestingly, the CCC complex is required for the hepatic surface expression of LRP1 [42], [43], supporting the hypothesis that this retromer-independent pathway could mediate insulin-dependent regulation of LRP1 trafficking via PID1. It remains to be determined which proteins specifically interact with PID1 to confer the perinuclear LRP1 retention.

Another important aspect of the present study is that knockout or knockdown of PID1 in hepatocytes *in vitro* and *in vivo* results in the constitutive presence of LRP1 at the plasma membrane. This cellular redistribution was, however, accompanied by a reduction of hepatocyte LRP1 levels, probably due to receptor shedding which has been described for this receptor [44]. Consistently, we observed that hepatic clearance of TRL and TRL remnants was diminished and plasma lipids were elevated in liver-specific *Pid1*^*−/−*^ mice in the absence of LDLR expression. In stark contrast, *Pid1* single knockout mice exhibited accelerated TRL uptake in the liver, which is explained by higher hepatic LDLR expression. A similar compensatory up-regulation of the LDLR has previously been observed by us in mice carrying a knockin mutation, which prevents the tyrosine phosphorylation of the distal LRP1 NPxY motif [16].

In conclusion, this study shows for the first time the relevance of PID1 for hepatic LRP1 function and lipoprotein metabolism. Our results indicate that reduction of PID1 expression in the liver, for example through siRNA, provides a novel therapeutic approach to increase LDLR activity to reduce the levels of circulating pro-atherogenic lipoproteins. From a cell biology perspective, it will be intriguing to follow the concept of retention adaptor proteins and to elucidate the detailed mechanism by which PID1 is able to retain LRP1 in a perinuclear recycling compartment.
